# Traumatic Brain Injury among Patients Admitted in Neurosurgical Unit in a Tertiary Care Centre: A Descriptive Cross-sectional Study

**DOI:** 10.31729/jnma.8197

**Published:** 2023-06-30

**Authors:** Ajit Shrestha, Nimesh Paudel, Ganesh Adhikari, Swikriti Shrestha, Sandesh Lamichhane, Subash Subedi, Neelam Jaishwal

**Affiliations:** 1 Department of Neurosurgery, Chitwan Medical College Teaching Hospital, Bharatpur, Chitwan, Nepal; 2 Chitwan Medical College Teaching Hospital, Bharatpur, Chitwan, Nepal; 3 Department of Emergency Medicine, Namaste Public Hospital, Damak, Jhapa, Nepal

**Keywords:** *contusion*, *craniotomy*, *traumatic brain injury*

## Abstract

**Introduction:**

Traumatic brain injuries are one of the leading causes of mortality, morbidity, and economic burden in context to Nepal. Non-contrast computed tomography is used for rapid diagnosis and repeat computed tomography helps to detect the progression and complications of cerebral injury. The aim of this study was to find out the prevalence of traumatic brain injury among patients admitted in the neurosurgical unit in a tertiary care centre.

**Methods:**

This descriptive cross-sectional study was conducted among patients admitted to the neurosurgery unit in the tertiary care centre from 1 August 2019 to 29 August 2020. Ethical approval was taken from Institutional Review Committee (Reference number: CMC-IRC/075/076-156). Convenience sampling method was used. Point estimate and 95% Confidence Interval were calculated.

**Results:**

Among 350 patients admitted in the neurosurgical unit, the prevalence of traumatic brain injury was 140 (40%) (34.87-45.13, 95% Confidence Interval). Change in management was required among 28 (20%) when computed tomography was done within 12-24 hours. Among them, 27 (19.29%) underwent surgical intervention after a repeat computed tomography scan.

**Conclusions:**

The prevalence of traumatic brain injury was found to be higher than similar studies done in similar settings.

## INTRODUCTION

Traumatic brain injury (TBI) is defined as a sudden insult to the brain caused by an external mechanical force that is non-degenerative and non-congenital and may result in an impairment of cognitive, physical, and psychosocial function and an altered state of consciousness.^1,2^ Trauma remains one of the leading causes of death and disability globally accounting to 9% of total mortality in Nepal.^3,4^

Prompt recognition of treatable injuries is critical to reducing mortality. In the acute setting, non-contrast computed tomography (CT) is the cornerstone for rapid diagnosis as it quickly and accurately identifies intracranial haemorrhage that warrants neurosurgical evacuation. Follow-up assessment using a CT scan is frequently necessary to detect the progression and stability of the lesion and evidence of delayed complications and sequels of cerebral injury, which can determine whether surgical intervention is necessary.^5^ However, patients who initially presented with head trauma often receive repeat CT scans in order to rule out the progression of their head injury.^6^

The aim of this study was to find out the prevalence of traumatic brain injury among patients admitted in the neurosurgical unit in a tertiary care centre.

## METHODS

A descriptive cross-sectional study was done among patients admitted in the neurosurgical unit with TBI, undergoing serial CT scans in the neurosurgical unit in a tertiary care centre. The study was conducted from 1 August 2019 to 29 August 2020 after obtaining ethical approval from the Institutional Review Committee of Chitwan Medical College Teaching Hospital (CMCTH) (Reference number: CMC-IRC/075/076-156). Patients with TBI who were subjected to two or more CT scans and gave written consent were included in this study. Patients who were intervened surgically based upon the findings of the first CT scan of the head, TBI less than 5 years of age, patients who expired or were discharged after the first CT head and patients on antiplatelet/anticoagulation therapy were excluded from the study. Data was collected using a selfstructured proforma. Convenience sampling method was used. The sample size was calculated using the formula:


$$\begin{array}{ll} \text{n} & ={\text{Z}}^{2}\times \frac{\text{p}\times \text{q}}{{\text{e}}^{\text{2}}}\\ & =1.96^{2}\times \frac{0.50\times 0.50}{0.10^{2}}\\ & =96 \end{array}$$

Where,

n = minimum required sample size
Z = 1.96 at 95% confidence interval (CI)
p = prevalence taken as 50% for maximum sample size calculation
q = 1-p
e = margin of error, 10%

The minimum sample size calculated was 96. However, the final sample size taken was 350.

The first CT scan (CT1) of the head was taken as soon as possible after head trauma-CT1 or admission CT scan. The second CT scan (CT2) was done 12-24 hrs after presentation/admission or any time if the patient showed neurological deterioration during the course of management. The third CT scan (CT3) was done 72 hrs after presentation/admission (if CT1 and CT2 donot lead to surgical management and the intracranial lesion is expected to increase) or any time if the patient shows neurological deterioration during the course of management. The findings of serial CT2 and CT3 were analyzed on the basis of the radiological progression of the hematoma which was judged by the treating neurosurgeon along with the radiologist and the management was done according to the hospital protocol for management. The proforma was prepared including the patient's details of age, sex, time of injury, mode of injury, the interval between trauma and CT imaging, and the Glasgow coma scale (GCS) at the time of presentation to the emergency room. Informed consent from the patient's family was taken. CT findings as recorded by the attending neurosurgeon included type of brain injury, presence or absence of intracranial hematoma, site and the number of intracranial lesions, mass effect/brain oedema, and presence of hypoxic changes. The outcome was based on changes in the repeat CT scans that had influenced management protocol. The outcomes were recorded as

1. Conservative: Observation with clinical monitoring
2. Medical management: Lower the intracranial pressure by osmotic diuretic (mannitol), 3% NaCl or hyperventilation (intubation), or decrease oedema by steroids.
3. Surgical management: Craniotomy and evacuation of intracranial hematoma or large contusion and decompressive craniotomy for mass effect.

Data were analysed using IBM Statistics SPSS 16. Point estimate and 95% CI were calculated.

## RESULTS

Among 350 patients admitted to the neurosurgical unit, the prevalence of traumatic brain injury was found to be 140 (40%) (34.87-45.13, 95% CI). Among 140 patients, 105 (70%) were male and 35 (25%) cases were female. All 140 (100%) patients underwent serial CT2 while only 69 (49.29%) cases underwent serial CT3. The median age of the patients with traumatic brain injury was 33 years. Most of the TBI were seen in road traffic accidents 89 (63.57%), followed by fall injuries 43 (30.71%), physical assault 6 (4.29%), and 2 (1.43%) included trauma due to animal attack.

**Figure 1:**
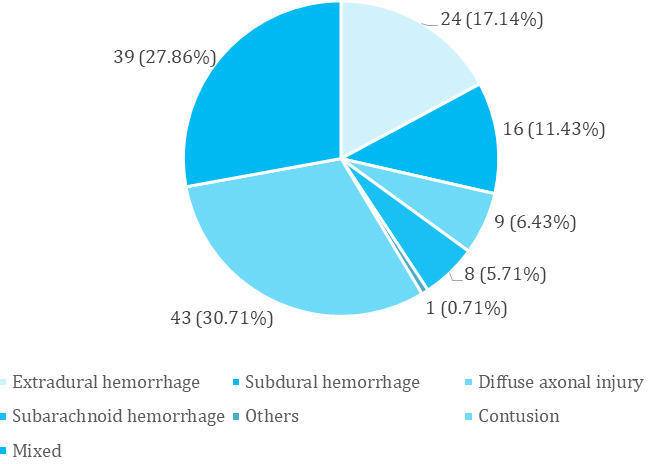
Types of lesions among patients with traumatic brain injury (n= 140).

The majority of the TBI lesions after CT1 were contusions 43 (30.71%), followed by mixed lesions 39 (27.86%) and EDH 24 (17.14%) (Figure 1).

The majority 122 (87.14%) of the cases underwent serial scan CT2 routinely. Only 18 (12.86%) cases were taken for CT2 based on neurological deterioration. Of the 69 (49.29%) cases undergoing serial CT3, 68 (48.57%) cases were routinely performed. Only 1 (0.71%) case was taken for CT3 based on neurological deterioration that was managed surgically.

Impression was categorized based on increasing, static, or decreasing hematoma size. Among 140 patients, 54 (38.57%) patients had increasing hematoma size on serial CT2 scan with 23 (16.43%) decreasing volume and 63 (45%) with static CT scan findings. A total of 69 (49.29%) patients underwent serial CT3 among which 67 (97.10%) had decreasing hematoma and 1 (1.45%) had increasing hematoma (Table 1).

**Table 1:** Radiological progression of intracranial lesions in serial CT among patients with traumatic brain injury (n= 140).

Progression/impression	(n %)
Increasing	54 (38.57)
Decreasing	23 (16.43)
No change	63 (45)

In the current study, 95 (67.86%) patients underwent CT2 within 12-24 hrs, 39 (27.86%) within 7-12 hrs, 4 (2.86%) cases after 24 hrs and only 2 (1.43%) within 6 hrs. Among these, change in management was required in those whose CT was done within 12-24 hr i.e. among 28 cases (29.47%).

Among 140 patients, 112 (80%) were managed conservatively and 28 (20%) were managed medically after an initial CT scan. The cases undergoing surgical intervention after repeat CT scan was 27 (19.28%) (Table 2).

**Table 2:** Management after CT1 among patients with traumatic brain injury (n= 140).

Initial management after CT-1	n (%)
Conservative	112 (80)
Medical	28 (20)

In scans based on routine CT, 18 (12.86%) of routine CT cases underwent surgical management. Among those 17 (94.44%) cases had undergone a change in management based on the finding in serial CT scans after neurological worsening. A total of 9 (50%) cases were taken for emergency craniotomies (Table 3).

**Table 3:** Type of management and CT2 among patients with traumatic brain injury (n= 140).

Management	Variables		Total n (%)
	Routine CT n (%)	Clinical worsening n (%)	
Conservative	64 (45.71)	1 (0.71)	65 (46.43)
Medical	40 (28.57)	8 (5.71)	48 (34.29)
Surgical	18 (12.86)	9 (6.43)	27 (19.29)
Total	122 (87.14)	18 (12.86)	140 (100)

Among patients undergoing serial CT3, hematoma size was decreasing among 68 (98.55%) patients. Only 1 (1.45%) had an increase in the size of the hematoma, which underwent a change in management from conservative to surgical management. The serial CT was done at 48 hrs from the initial CT1 due to neurological deterioration that showed increasing hematoma and the patient underwent surgical management (Table 4).

**Table 4:** Management after CT-3 among patients with traumatic brain injury (n= 69).

Type of management	n (%)
Conservative	42 (60.87)
Medical	27 (39.13)

## DISCUSSION

In our study, the prevalence of traumatic brain injury among patients admitted to the neurosurgical unit was found to be 140 (40%) which was higher as compared to a study where the prevalence of head injury was reported to be 31.06%.^7^ Because early identification of worsening brain injury may allow for more rapid and early intervention, patients with documented intracranial injuries often undergo frequent routine serial scans given that significant radiological changes may occur with minimal or no clinical and neurological changes.^8^ The major goal 'detection before deterioration' and timely intervention is the key to serial CT scans.^5^ This study provides light on the prevalence of traumatic brain injury, the timing of serial CT scans performed and the identification of hematoma progression.

The majority of these cases underwent CT scans routinely. Within 6 hrs most of the cases were clinically static and beyond 24 hrs only 1 case had changed in management. Likewise, the majority of the cases with clinical worsening had undergone serial CT scans within a 7-24 hrs period. Of 18 cases with clinical worsening, 9 underwent CT scans within 7-12 hrs and 7 cases within 13-24 hrs. Similar study done in India recommended initial CT scan could be followed by a second CT scan within 24 to 48 hrs for the detection of evolving lesions and change in management. The study reported that 75% of the cases who went for surgical management based on the findings of CT2, had CT1 done less than 6 hrs after the injury which was similar to the findings of our study.^8^

In the current study, 38.57% of cases had progression of hematoma with 16.43% decreasing and 45% remaining static. In CT3 almost all cases had resolving hematoma. One of 69 cases had undergone CT3 due to neurological deterioration (oedema) which was managed surgically. In a similar study, 35% of scans showed the progression of hematoma, 43% of scans showed no changes and 22% of cases showed decreasing hematoma.^9^ In their study, 81% of scans were routinely done without evidence of neurological deterioration. Likewise, another study reported similar findings with the progression of hematoma in 33.3% of cases within 48 hrs and the incidence of increasing hematoma was predominant in EDH and contusions, either alone or as mixed lesions.^8^ Repeat CT scans demonstrated lesions earlier than clinical deterioration in a study and the likelihood of significant growth of hematoma increases on subsequent scans.^10,11^ This shows that the progression of hematoma is an inevitable process and timely CT scans can diagnose the expanding hematoma before neurological deterioration.

In this study, 51 (36.43%) of initial 112 (80%) of patients were managed conservatively and 20 (14.29%) of cases of initial 28 (20%) patients managed medically underwent a change in management after CT2. Surgical intervention was done in overall 27 (19.29%) of cases after CT2. Change in management to medical intervention was seen overall in 34.3% of cases after CT2. However, only one case had undergone a change in management after serial CT3. In a retrospective study, 17% of patients underwent subsequent neurosurgical intervention which is similar to the findings of our study.^12^ Another study reported that overall, 32% of patients with progression of injury on repeated CT underwent one or more changes in nonsurgical management which are comparable to this current study.^13^ Likewise, a prospective study showed that changes in management decisions were seen in as many as 47 (23%) of cases based upon findings of repeat CT that doesn't corroborate the current study.^8^ However, the incidence of surgical intervention was seen among 14% of the patients after CT2 and a further 9.7% of cases undergoing surgical intervention after CT3, within 48 hours of trauma which is similar to our present study.

A retrospective study suggested that a routine repeat CT scan within 24 hrs after blunt head trauma might minimize the potential neurological deterioration in patients with GCS lower than 12.^12^ In their study, 161 (95.83%) patients underwent routine repeat CT while 7 (4.17%) cases had repeat CT due to worsening neurological conditions which was similar to our study where 122 (87.14%) underwent routine repeat CT and 18 (12.86%) had repeat CT due to worsening neurological conditions. Of 28 surgically treated cases, 6 (10%) had neurological worsening while the rest had routine CT scans. This finding is similar to the results of our study as out of 27 surgically managed cases, 9 cases (50%) had clinical worsening based on CT2 findings. However, we couldn't categorize the patients based on GCS and document the improvement accordingly.

A similar study concluded that all patients with a head injury should undergo CT after neurological deterioration because it leads to intervention in over one-third of the patients.^9^ Another similar study supported a selective approach for repeating head CTs with emphasis on changes in neurological symptoms and GCS.^14^ In another study it was suggested that a repeat CT scan may be preferred only in the presence of clinical worsening and when CT1 is done within 2 hr after trauma.^15^

The limitation of our study is that the study was carried out among a small population and in a single centre, thus lacks the generalizability of the outcomes.

## CONCLUSIONS

The prevalence of traumatic brain injuries was found to be higher than similar studies done in similar settings. However, scans indicated after neurological deterioration almost always resulted in interventional changes whether surgical or medical. Timely performed serial CT scans are important in changing management of the patients with traumatic brain injury and serve to decrease the risk for neurological deterioration by detecting radiological progression prior to clinical worsening.
